# Supplementary material for: Selenium and Episodic Memory: The Moderating Role of Apolipoprotein E ε4
Source: Nutrients. 2025 Feb 6;17(3):595. doi: 10.3390/nu17030595 (PMC11819958; doi:10.3390/nu17030595)
Supplement: Supplementary file 1 [file nutrients-17-00595-s001.zip › nutrients-3463830-supplementary.pdf]

Supporting Online Content

Table S1 Results of multiple linear regression analyses that included interaction terms for the association between serum Se levels and APOE4-positivity in predicting non-memory cognitive decline

Table S2 Results of the multiple linear regression analyses of the association between the serum Se levels and cognitive decline according to APOE4 subgroup in MCI older adults (n = 113)

Table S3 Results of the multiple linear regression analyses of the association between the serum Se levels and cognitive decline according to APOE4 subgroup in older adults without a 3-month decline in food intake (n = 182)

Table S1 Results of multiple linear regression analyses that included interaction terms for the association between serum Se levels and APOE4-positivity in predicting non-memory cognitive decline

	B	*95% CI*	*P*
**Verbal fluency (**for executive function/attention/language)			
Se levels	0.019	-0.004 to 0.042	0.108
APOE4-positivity	0.051	-2.032 to 2.134	0.962
Se levels ×APOE4-positivity	0.001	-0.018 to 0.021	0.881
**Modified Boston naming test (**for language)			
Se levels	0.012	-0.004 to 0.028	0.143
APOE4-positivity	0.671	-0.679 to 2.221	0.328
Se levels ×APOE4-positivity	-0.004	-0.017 to 0.009	0.541
**Constructional praxis (**for visual spatial/perceptual skill)			
Se levels	-0.002	-0.013 to 0.009	0.667
APOE4-positivity	0.785	-0.148 to 1.717	0.099
Se levels ×APOE4-positivity	-0.006	-0.015 to 0.002	0.151

Abbreviations: Se, selenium; APOE4, apolipoprotein ε4 allele; EMS, episodic memory score.
To explore the moderating effects of APOE4-positivity on the associations between serum selenium level and non-memory cognition, the multiple linear regression analyses were performed including two-way interaction terms between selenium level and cognition as additional independent variables.

Table S2 Results of the multiple linear regression analyses of the association between the serum Se levels and cognitive decline according to APOE4 subgroup in MCI older adults (n = 113)

	B	*95% CI*	*P*
**EMS**			
Overall			
Model 1	0.096	0.031 to 0.160	0.004
Model 2	0.099	0.030 to 0.169	0.005
APOE4-negative			
Model 1	0.097	0.033 to 0.161	0.004
Model 2	0.100	0.028 to 0.171	0.007
APOE4-positive			
Model 1	0.083	-0.179 to 0.345	0.513
Model 2	-0.003	-0.341 to 0.335	0.984
**TS**			
Overall			
Model 1	0.165	0.063 to 0.267	0.002
Model 2	0.171	0.062 to 0.279	0.002
APOE4-negative			
Model 1	0.171	0.064 to 0.277	0.002
Model 2	0.171	0.053 to 0.288	0.005
APOE4-positive			
Model 1	0.087	-0.280 to 0.455	0.622
Model 2	0.001	-0.462 to 0.462	0.999

Abbreviations: Se, selenium; APOE4, apolipoprotein E ε4 allele; EMS, episodic memory score; TS, total score of the Consortium to Establish a Registry for Alzheimer's Disease; VRS vascular risk score.
The first model included age, sex, APOE4, VRS, and education as covariates; the third model included those covariates plus protein intake, fruit/vegetable, albumin, fasting glucose, and HDL- or LDL-cholesterol.

Table S3 Results of the multiple linear regression analyses of the association between the serum Se levels and cognitive decline according to APOE4 subgroup in older adults without a 3-month decline in food intake (n = 182)

	B	*95% CI*	*P*
**EMS**			
Overall			
Model 1	0.062	0.018 to 0.106	0.006
Model 2	0.066	0.020 to 0.112	0.005
APOE4-negative			
Model 1	0.065	0.020 to 0.109	0.005
Model 2	0.071	0.023 to 0.119	0.004
APOE4-positive			
Model 1	0.033	-0.111 to 0.177	0.640
Model 2	-0.009	-0.200 to 0.182	0.924
**TS**			
Overall			
Model 1	0.119	0.048 to 0.191	0.001
Model 2	0.123	0.047 to 0.199	0.002
APOE4-negative			
Model 1	0.065	0.020 to 0.109	0.005
Model 2	0.071	0.023 to 0.119	0.004
APOE4-positive			
Model 1	0.033	-0.111 to 0.177	0.640
Model 2	-0.009	-0.200 to 0.182	0.924

Abbreviations: Se, selenium; APOE4, apolipoprotein E ε4 allele; EMS, episodic memory score; TS, total score of the Consortium to Establish a Registry for Alzheimer's Disease; VRS vascular risk score.

The first model included age, sex, APOE4, VRS, education, and clinical diagnosis as covariates; the third model included those covariates plus protein intake, fruit/vegetable, albumin, fasting glucose, and HDL- or LDL-cholesterol.
